# A toxicological perspective on climate change and the exposome

**DOI:** 10.3389/fpubh.2024.1361274

**Published:** 2024-04-08

**Authors:** Robert Barouki

**Affiliations:** Université Paris Cité, INSERM U 1124 (T3S), Paris, France

**Keywords:** adverse outcome pathway, xenobiotics, heat, air pollution, hallmarks of diseases

## Abstract

Climate change is accompanied by changes in the exposome, including increased heat, ground-level ozone, and other air pollutants, infectious agents, pollens, and psychosocial stress. These exposures alter the internal component of the exposome and account for some of the health effects of climate change. The adverse outcome pathways describe biological events leading to an unfavorable health outcome. In this perspective study, I propose to use this toxicological framework to better describe the biological steps linking a stressor associated with climate change to an adverse outcome. Such a framework also allows for better identification of possible interactions between stressors related to climate change and others, such as chemical pollution. More generally, I call for the incorporation of climate change as part of the exposome and for improved identification of the biological pathways involved in its health effects.

## Introduction

Health and wellbeing (Goal 3) and climate action (Goal 13) are 2 of the 17 sustainable development goals (SDGs) formulated and endorsed by the United Nations. A number of other SDGs refer to environmental quality. Despite the literature on human health and climate change building and evolving since at least 1989, the Conferences of the Parties (COPs) on climate change (henceforth CC) have barely mentioned health. However, during COP28 (held in December 2023), extensive discussions of the impact of CC on health took place. Indeed, health is mentioned in the draft statement: “Attaining resilience against climate change related health impacts, promoting climate-resilient health services, and significantly reducing climate-related morbidity and mortality, particularly in the most vulnerable communities”.^1^ A better understanding of the impact of CC on health and wellbeing is increasingly recognized as critical for public health. In this perspective article, the relevance of the exposome concept and toxicological tools to improve understanding of the relationship between CC and health is discussed.

Christopher Wild coined the term exposome in 2005 and defined it as the life-course environmental exposures from the prenatal period onward (1). These exposures include chemical, biological, and physical, as well as social inequalities and psychosocial influences. In that sense, the exposome is the complement of the genome (2). This definition was further developed by Rappaport and Smith, who highlighted the relevance of a thorough analytical characterization of chemicals in body fluids. This corresponds to the internal component of the exposome, while environmental and behavioral factors correspond to the external components of the exposome (3). Miller and Jones included the biological impacts of exposures in their definition of the exposome (4). Consistent with this, it was recently suggested that toxicological tools could be useful for the characterization of the human exposome (5).

The effects of anthropogenic CC on human health correspond to an increasingly visible change in the exposome (6, 7). CC influences exposures to physical stressors (heat and UV), chemical stressors (ground-level ozone and particles), and biological stressors (vectors and the diseases they transmit, infectious agents in water, and pollens), as well as psychosocial stressors induced by extreme weather events (7). The exposome concept appears well suited to analyze these effects by incorporating all these stressors as well as their interactions (1). Furthermore, the “co-benefits” strategies for CC mitigation call for a holistic assessment of the attenuation of pollution sources (e.g., increased active transport has been suggested as reducing greenhouse gas emissions while also improving fitness) and are in line with the exposome concept (8, 9).

Several recent reviews have highlighted the relevance of toxicological tools and concepts for the assessment of exposome health effects (4, 5, 10). Indeed, toxicological studies are currently based on omics (large-scale data-rich studies), systems biology, predictive molecular tools, computational approaches, and the adverse outcome pathway (AOP) framework in addition to more traditional experimental approaches (11, 12). Furthermore, there is a growing interaction between toxicology, epidemiology, and exposure sciences that is critical for risk assessment. With this in mind, it is relevant to explore whether insights from toxicology can improve the understanding of the health effects of CC, including through an exposome “lens.”

## Altering the external component of the exposome

**Figure 1:**
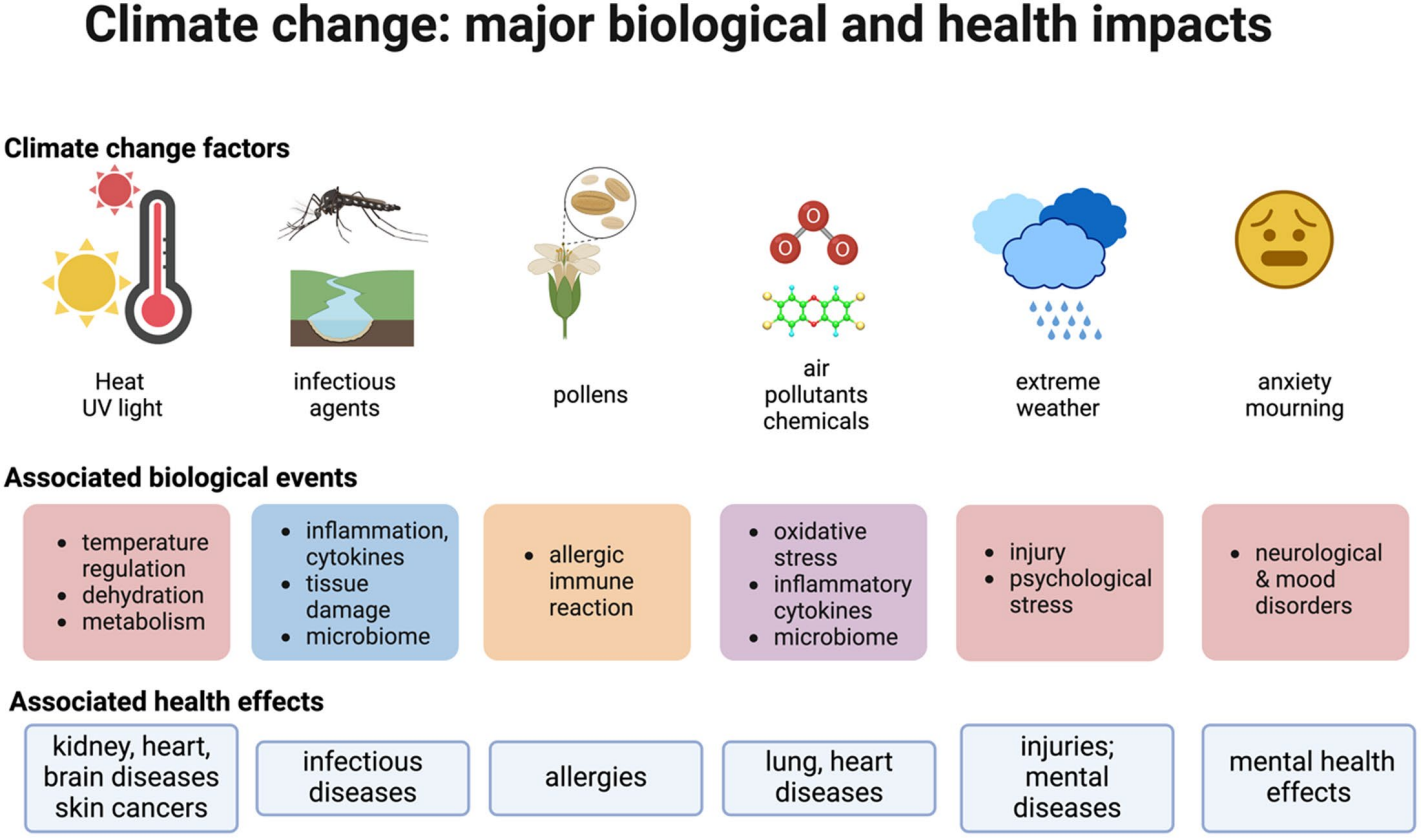
Climate change: major biological and health impacts. Climate change modifies a variety of environmental factors. These may interact with other stressors, thus altering a number of biological events and ultimately leading to health effects. The figure highlights key biological events as a first step in using the adverse outcome pathways framework. The figure was drawn with Biorender.

CC alters several forms of exposure (see Figure 1). CC impacts the amounts and distribution of several environmental factors, which may lead to biological and health outcomes. In order to analyze these effects, modifications in environmental stressors induced by CC in different matrices are discussed below. The two major matrices that are analyzed are air and water, and the contributions of factors modified by CC as well as those of other environmental determinants such as pollution are examined.

One of the consequences of CC is the increase in ground-level ozone (13–15). Ozone is produced from chemicals of natural- and human-derived origin, such as fossil fuel combustion. Ozone is a known pulmonary toxicant; its effects can combine with other air pollutants to increase the risk of lung diseases (16). A CC-associated increase in drought will also increase atmospheric particulate matter. There are significant differences in the nature of particles that increase with CC (dust, sand, and smoke from wildfires) as compared to traffic, and it is likely that such increases will have systemic human health effects (17). Dust and sand particles can bind a variety of chemicals, sometimes transporting them over long distances. Wildfires will also increase pollutants, with considerable effects (7). Pollens and allergens will also increase with CC, and this will impact a number of respiratory and other diseases (18). Pollens may interact with infectious agents and other air pollutants to exacerbate diseases. Air quality will be considerably altered by CC in many areas of the world, particularly those where drought is expected to increase. On the other hand, any substantial reduction in the combustion of coal and other fossil fuels (to slow the rate of CC) will act to counter such a deterioration in air quality. Several environmental factors that are modified by CC elicit biological events such as inflammation, oxidative stress, and immune dysregulation, ultimately leading to lung and other diseases.

The decrease in water availability and quality due to drought will lead to increased concentrations of chemicals for humans and other species (19). Another critical factor is the increasing number of extreme events and floods. These spread infectious agents as well as potentially toxic chemicals, degrading water and soil quality (20). Increased heat, another important CC effect, may alter the properties of chemicals to which people are exposed, including their solubility, persistence, and volatility (19). However, at this stage, it is difficult to draw a general conclusion about whether the effect of heat on chemical toxicity is negative or positive. Similarly, soil quality will depend on the physical and metabolic properties of chemical contaminants.

## Changes in the internal component of the exposome: xenobiotic toxicokinetics

Changes in the external exposome can lead to changes in the internal exposome. The latter can also be modified if the absorption, distribution, metabolism, and elimination of exogenous chemicals (xenobiotics) are altered by CC. There is indirect evidence that this may be the case. The impact of CC may vary depending on the type of xenobiotics. Some xenobiotics, such as persistent organic pollutants (POPs), are not metabolized and are persistent in the body, while others are readily metabolized and eliminated. Concerning POPs, which are stored in human adipose cells (21), there are suggestions that global warming may contribute to increased obesity and thus increased storage capacity (22). Whether this could lead to increased health effects from these chemicals is unclear.

Xenobiotic metabolic pathways are influenced by changes in physiological states, which may be altered by CC. Xenobiotic metabolism and elimination are primarily dependent on the functional liver, gut, and kidney. Heat, especially when accompanied by dehydration, alters renal function and impairs other metabolic organs, leading to accumulated levels of toxins (23, 24). Another effect of CC may be an increased risk of hepatic infection and inflammation, potentially also harming xenobiotic metabolism (25).

The microbiome is also sensitive to CC possibly leading to modifications in the absorption of chemicals (26, 27).

## Common health targets of chemicals and climate change-related stressors

Several health effects of CC arise from increased exposure to environmental pollutants. Examples of such health impacts are detailed below.

### Immunotoxicity and infections

A hallmark of CC is an altered distribution (in some cases increased) of infectious diseases. This is due to changes in vector locations, accelerated life cycles of pathogens within some vectors, and pathogens contaminating water. Many chemicals have been shown to interfere with the immune system, leading in some cases to immunosuppression (28). This is in particular the case of dioxins and poly- and perfluoroalkyl substances (PFASs). Higher concentrations of PFAS correlate with decreased vaccination responses in children and an increased risk of infection (29). In the case of dioxin-like compounds, the mechanisms of immunotoxicity appear to be linked to the immune functions of the dioxin receptor (aryl hydrocarbon receptor), in particular in barrier organs (e.g., gut and skin) (30). Furthermore, both dioxin-like compounds and PFAS are highly persistent chemicals and will remain contaminants of high concern in the next decades, even if their global production is rapidly regulated (PFAS) or limited (dioxins). It is not proven yet that immunotoxicants will affect CC-associated infectious agents, but this is biologically plausible.

### Neurotoxicity and climate change

Many chemicals have been proven to be likely or proven neurotoxicants (31, 32). The two main outcomes are developmental and adult neurotoxicity, in particular neurodegenerative diseases. There are several possible interactions between neurotoxicants and CC. Neuronal oxidative stress occurs in neurodegenerative diseases (33). Some of the health impacts of CC are also partly mediated by oxidative stress; thus, these consequences could be additive or synergistic. Furthermore, excessive heat and dehydration may also cause neurological harm. Further study of neurotoxins, including their interactions with infectious agents and air pollution that may also be altered in their risk profile due to CC, is of importance.

### Mental health

It is now accepted that CC can lead to a range of mental health effects, including those resulting from exposure to extreme weather events (34). It is plausible that these conditions may interact with chemical exposure, aggravating or generating a variety of neurocognitive diseases, including among children.

### Pulmonary and cardiac toxicity

Air pollution is generated by traffic, industry, and agriculture and is likely to be increased by CC (16). This eventually leads to lung and heart diseases. Importantly, heat also contributes to deleterious effects on these organs.

### Reproductive health

Recent evidence has suggested that CC-associated pathways harm reproductive health, via means such as air pollution, exposure to wildfire, and excessive heat (35). Different mechanisms are involved, depending on the nature of the stressor. Many chemicals are also known to lead to reprotoxicity, in particular, endocrine disruptors (36, 37).

### Cancer

An increased risk of cancer associated with CC is plausible because of increased exposure to UV and some chemicals via the pathways discussed above (9). The actual impact is, at this stage, difficult to assess. An increase in infectious agents could also lead to increased cancer.

These examples indicate that CC and chemical agents have common health impacts. It is still unclear if the interaction between these stressors is additive, synergistic, or otherwise. In some cases, interactions are biologically plausible (e.g., immunosuppressants and infectious agents), but in other cases, this remains speculative. An improved understanding of toxicology could help better characterize these interactions.

## Relevance of new frameworks in toxicology to climate change impacts

In recent years, several new frameworks have been put forward in biomedical research, including toxicology. Among these, disease “hallmarks” (e.g., DNA integrity as an indicator of cancer) highlight major biological processes perturbed by illness (38, 39). Key characteristics are focused on the agents that can lead to an adverse outcome and are mostly used in the field of cancer (e.g., genotoxicants) (40). The characterization of AOPs is currently one of the major objectives in toxicology. An international effort has been launched to identify such pathways and publish them on a dedicated website, AOPwiki.^2^ An AOP is a chain of linked biological events that begins with an identified molecular trigger, ultimately leading to an adverse outcome (41). Importantly, event–event relationships are evidence-based. Each of these frameworks is useful under certain conditions; they are complementary to a large extent.

Concerning CC and its possible interaction with other exposome factors, I argue that the AOP framework is relevant. It is noteworthy that AOPs are agnostic in that they are not specific to a stressor but rather can be triggered by a variety of them. A first step would be to identify biological events that link CC to health effects. This allows us to link biological events that are included in available AOPs to CC-related environmental stressors or prompt the development of additional AOPs. Furthermore, AOP networks, which illustrate how different AOPs share common events, can show how CC-related stressors interact with other stressors. For example, increased inflammatory cytokines can be key events in pathways triggered by CC-related stressors as well as by chemical contaminants (e.g., the key event n°1,496: “increased secretion of inflammatory mediators,” AOPwiki) (see Figure 1). Oxidative stress is elicited by chemical, physical, and psychological stressors (42). Extreme heat is a physical stressor that leads to a variety of life-threatening outcomes and targets different organs including the kidney, heart, and brain (7). It initially leads to a loss of internal temperature control, which is then associated with different outcomes. Developing AOPs, including the dysregulation of body temperature control and ultimately leading to diverse health outcomes, would be useful to identify links to available AOPs and to infer possible interactions with other stressors.

In a previous commentary, we called for the application of the AOP framework to social hazards (43). Most of the effort in the field of AOPs has been accomplished by toxicologists using data primarily derived from studies on chemical hazards. However, since AOPs are agnostic by design, it should be possible to use the framework for a variety of stressors and conditions reflecting the universality of the concept. An advantage of such an approach is that it allows a better description of interactions between different types of stressor-elicited pathways (e.g., social and physical, CC, and chemical). We argued this should bridge different fields to the benefit of biomedical and environmental research.

## Conclusion and recommendations

There is increasing knowledge of the impact of CC on human health; however, much remains unknown. Delineating the mechanistic pathways will help to identify and predict possible interactions between different stressors associated with CCs and other environmental effects. A toxicological approach could be useful.

The main recommendation is to further explore the relevance of the AOP framework in the context of CC. This requires identifying the current AOPs or key events in AOPwiki that may be relevant for CC effects, for example, inflammatory cytokines, oxidative stress, and skin sensitization (see text footnote 2sssssw). Such AOPs may indicate possible interactions between CC and other stressors. For some CC factors, such as heat, it may be useful to develop new AOPs that can also be used to identify possible interactions.

Another related proposal would be to systematically look for interactions between different environmental factors and CC effects. This would support the identification of vulnerable individuals, problematic co-exposures, or possible antagonistic effects. For example, it would be interesting to determine the interactions between traffic- and industry-related air pollution and infectious diseases elicited by CC; indeed, the immune and inflammatory effects elicited by air pollution could interfere with the normal response to infections. Another example is the interaction between increased ozone and air pollution and lung diseases.

A specific focus on the effect of CC on children–particularly those sensitive to environmental stressors–is also recommended. The exposome concept highlights life-course effects, with childhood being a particularly vulnerable stage of development, in particular for neurocognitive functions. The combination of different stressors associated with CC (e.g., heat waves, extreme weather events, wildfires, and anxiety) may have a particularly detrimental impact on children.

## Data availability statement

The original contributions presented in the study are included in the article/supplementary material, further inquiries can be directed to the corresponding author/s.

## Author contributions

RB: Conceptualization, Formal analysis, Funding acquisition, Supervision, Validation, Writing – original draft, Writing – review & editing.

## Footnotes

^1^https://unfccc.int/sites/default/files/resource/cma2023_L17_adv.pdf

^2^https://aopwiki.org/
